# Dynamic analysis of weak prisoner’s dilemma game considering conservative and aggressive cooperative strategy

**DOI:** 10.1371/journal.pone.0354591

**Published:** 2026-07-24

**Authors:** Sihui Li, Sida Kang, Hongyu Liu

**Affiliations:** School of Business Administration, University of Science and Technology Liaoning, Anshan, Liaoning, China; Teesside University, UNITED KINGDOM OF GREAT BRITAIN AND NORTHERN IRELAND

## Abstract

The balancing of benefits is a critical pathway for maintaining cooperative relationships among selfish individuals within a group. Relevant research has confirmed that the ultimate outcomes of altruistic actions essentially revert to the level of self-interest. This study posits that individuals exhibit heterogeneous cooperative behaviors in social interactions due to differences in cognitive abilities and personality traits; accordingly, conservative cooperative strategy and aggressive cooperative strategy are introduced. Conservative cooperators are characterized by incurring additional identification costs when interacting strategically with other individuals, whereas aggressive cooperators demonstrate a higher level of resource investment to promote the emergence of cooperation. The results indicate that, in infinite well-mixed populations, reducing identification cost and increasing cooperative benefit can, to some extent, promote cooperation. In structured populations, especially under the random neighbor unconditional imitation rule, the relative advantages of the three strategies increase or decrease in a stage-dependent manner as the identification cost and aggressive cooperation cost vary. The research findings confirm that the presence of differentiated cooperative strategies effectively sustains group cooperation, providing new theoretical insights and model support for analyzing the mechanisms of cooperation evolution in real-world scenarios.

## Introduction

Throughout the long course of biological evolution, cooperative behavior has remained an intriguing and enduring puzzle. Worker bees in colonies sacrifice individual reproductive opportunities to serve the collective, and African wild dogs achieve a high hunting success rate through cooperative group action that far exceeds that of solitary individuals. These phenomena, which appear to contradict the principle of “survival of the fittest,” have nonetheless emerged through long-term evolutionary processes and represent strategic choices that balance individual interests with group persistence [1,2]. Cooperative behavior is likewise of fundamental importance in human societies. Individuals sometimes choose to forgo personal interests in favor of collective benefits; however, the phenomenon of sacrificing collective interests for self-interest is also quite common, giving rise to a range of social dilemma issues [3–6].

Evolutionary game theory provides a foundational analytical framework for resolving social dilemmas and for explaining the mechanisms through which individual cooperation emerges and is sustained [7]. For example, classical models including the Prisoner’s Dilemma, the Hawk–Dove game, and the Snowdrift game [8–10]. As research has progressed, these classical models have been further improved and elaborated. In 1988, Axelrod proposed an evolutionary cooperation model grounded in the Prisoner’s Dilemma, providing an in-depth analysis of how factors such as population size, strategy space, and payoff structure influence the evolution of cooperation [11]. In 2006, Nowak systematically identified five fundamental mechanisms underlying the evolution of cooperation, namely kin selection, direct reciprocity, indirect reciprocity, network reciprocity, and group selection [12,13]. Moreover, it has been suggested that cooperators’ reputation [14–17], dynamic social networks [18,19], heterogeneity among agents [20–22], and reciprocal reward mechanisms can significantly affect the maintenance of cooperative behavior [23–25].

In recent years, evolutionary game theory has found extensive applications in a wide range of fields, such as psychology, management, and medicine [26–29]. Drawing on the analytical frameworks and theoretical tools provided by game-theoretic models, researchers have systematically explored the formation logic, operating mechanisms, and optimization strategies of cooperative patterns, leading to the extension and practical implementation of traditional cooperation paradigms. In addition to the standard categories of cooperators and defectors, scholars have incorporated agent types such as quasi-cooperators [7], quasi-defectors [30], and defensive cooperators [31] into evolutionary cooperation models, and verified that their inclusion can promote the evolutionary emergence and stability of cooperation. In some practical settings, the selection of individual strategies is shaped by heterogeneity in risk preferences [32–34], capability endowments, and environmental perception [35,36].

From a definitional perspective, the previously mentioned quasi-cooperators and quasi-defectors are primarily weakened variants of pure cooperation and pure defection, representing differentiated agents with varying degrees of cooperative tendencies. Defensive cooperators aim solely to resist exploitation by defectors; their defensive behaviors are triggered only after encountering betrayal, and cooperation itself incurs no additional fixed costs. Their defense cost is passively triggered, following the core logic of cooperate first, defend upon betrayal. In contrast, the conservative cooperators and aggressive cooperators introduced in this paper are two novel cooperative strategies based on risk preference and proactive cost investment. Both strategies proactively evaluate the expected profitability of cooperation in advance based on their risk preferences to determine their respective levels of resource investment. Furthermore, during interactions, both must continuously bear their respective costs regardless of the opponent’s cooperative intentions. From a practical standpoint, quasi-cooperators and quasi-defectors typically exist in loosely structured organizations, representing irresolute individuals within a group. Defensive cooperators are mainly applicable to scenarios involving ex-post accountability. The conservative and aggressive cooperators proposed herein are more suited for organizations with robust operational mechanisms, particularly facilitating ex-ante decision-making in small and medium-sized enterprises. Based on this rationale, this paper constructs a three-strategy cooperative game model to investigate the evolutionary states of cooperation in both well-mixed populations and cellular automata-based structured populations.

The remainder of this paper is organized as follows. The model section provides a detailed description of the evolutionary game models, considering both infinite well-mixed populations and structured populations implemented via cellular automata. We then develop an evolutionary game model incorporating conservative cooperation, aggressive cooperation, and defection. The results section derives the stability conditions of the equilibrium points and characterizes the corresponding stable states. Numerical simulations are then conducted to illustrate the evolutionary dynamics of the three strategies in both infinite well-mixed populations and cellular-automaton-based structured populations. The paper concludes with a summary of the main findings.

## The model

### Infinite well-mixed population model

In the Prisoner’s Dilemma, cooperation and defection are the two fundamental strategies available to the players. When both players choose to cooperate, they each receive a reward *R*. If both players choose to defect, they each obtain a punishment *P*. When one player cooperates while the other defects, the defector receives the temptation benefit *T*, whereas the cooperator obtains the sucker’s benefit *S*. In this study, we focus on the weak Prisoner’s Dilemma by setting *S* = *P* = 0. The payoff structure satisfies the inequalities *T* > *R* > *P* > *S* and 2*R* > *T* + *S*. To ensure the strict inequality 2*R* > *T* + *S* holds, we set T=b∈(1,2). Under these conditions, defection emerges as the unique Nash equilibrium of the game [37,38].

Cooperators may exhibit conservative or aggressive characteristics due to heterogeneity in environmental resources and individual cognitive capacities. For individuals adopting a conservative cooperative strategy, a cautious attitude can mitigate potential losses when interacting with defectors. In contrast, individuals employing an aggressive cooperative strategy are typically able to enhance the benefits of cooperation through more efficient decision-making and increased resource investment. Accordingly, this study incorporates conservative cooperative strategy and aggressive cooperative strategy into the game-theoretic framework to construct the proposed model.

In this paper, the strategy choices of all individuals are classified into three categories: the conservative cooperative strategy (*IC*), the aggressive cooperative strategy (*MC*), and the defection strategy (*D*). Specifically, conservative cooperators (*IC*) generally refer to cautious, risk-averse cooperators whose primary focus is on risk control. Aggressive cooperators (*MC*) typically denote proactive cooperators who actively invest and aim to enhance collaborative returns. Meanwhile, defectors (*D*) are defined as non-cooperative individuals who profit by exploiting the contributions of others. Conservative cooperators possess a high level of risk identification ability and are required to incur an identification cost k(0≤k≤1) when engaging in a game with any individual in the population. When a conservative cooperator engages in a game with a defector, the loss suffered by the conservative cooperator is reduced, which is referred to as the conservative benefit n(0≤n≤1). while the benefit of the defector is correspondingly decreased by *n*. During the game process, aggressive cooperators invest a greater amount of resources and therefore incur an aggressive cooperation cost a(0≤a≤1) when interacting with any individual in the population. When an aggressive cooperator interacts with another cooperator, the cooperative benefit is affected by the type of the counterpart. If the opponent is a conservative cooperator, the additional resource investment by the aggressive cooperator leads to an increase of c(0≤c≤1) in the cooperative benefit for both players. If the opponent is also an aggressive cooperator, a higher benefit is obtained, referred to as the aggressive benefit β(0≤β≤1). When an aggressive cooperator interacts with a defector, the increased resource investment results in a larger loss for the aggressive cooperator, quantified by m(0≤m≤1), while the benefit of the defector is correspondingly increased by *m*. Let R=1,S=P=0,T=b∈(1,2), the payoff matrix is given as follows:


$$\begin{array}{c} \;\begin{array}{c} \begin{array}{ccc} \;IC & \;MC & \;D \end{array}\\ \begin{array}{c} IC\\ MC\\ D \end{array}\left(\begin{array}{ccc} 1-k & 1+c-k & n-k\\ 1+c-a & 1+\beta -a & -a-m\\ b-n & b+m & 0 \end{array}\right) \end{array} \end{array}$$ (1)

The parameter definitions for System 1 are shown in Table 1.

**Table 1:** The parameter description of System 1.

Parameter	Description
*k*	The identification cost of conservative cooperators.
*n*	Benefit to conservative cooperators and loss to defectors in an encounter.
*a*	The cost of aggressive cooperation for aggressive cooperators.
*c*	Cooperation payoff between aggressive cooperators and conservative cooperators.
β	Aggressive gain from interactions among aggressive cooperators.
*m*	Loss to aggressive cooperators and gain to defectors in an encounter.

We consider an infinite well-mixed population in which the above game is played. Let *x*, *y* and z=1−x−y denote the fractions of conservative cooperators, aggressive cooperators, and defectors in the population, respectively. Individuals are allowed to interact with any other individual in the population. Accordingly, the average benefits of the three strategies are given by:


$$\begin{array}{c} \;\{\begin{array}{ccc} P_{IC} & = & (1-k)x+(1+c-k)y+(n-k)z,\\ P_{MC} & = & (1+c-a)x+(1+\beta -a)y-(a+m)z,\\ P_{D} & = & (b-n)x+(b+m)y. \end{array} \end{array}$$ (2)

The corresponding replication equations are obtained as follows:


$$\begin{array}{c} \;\{\begin{array}{c} \dot{x}=\frac{dx}{dt}=x(P_{IC}-\bar{P}),\\ \dot{y}=\frac{dy}{dt}=y(P_{MC}-\bar{P}),\\ \dot{z}=\frac{dz}{dt}=z(P_{D}-\bar{P}). \end{array} \end{array}$$ (3)

The expected benefit of the entire population is denoted by $\bar{P}=xP_{IC}+yP_{MC}+zP_{D}$.

### Structured population model

To investigate the role of spatial structure in the evolutionary dynamics of cooperation, this study introduces a Cellular Automaton (CA) model to characterize structured populations. Within this modeling framework, each individual corresponds to an independent node in the network and engages in game interactions exclusively with its neighboring nodes. Specifically, a two-dimensional square lattice of size L×L is employed as the interaction network, resulting in a total population size of *N* = *L*^2^. Each node irepresents an individual, where x,y∈1,2,...,L. The Moore neighborhood is adopted to define local interactions, such that each individual interacts with its eight nearest surrounding neighbors. To eliminate boundary effects that may bias the simulation results, periodic boundary conditions are applied. Accordingly, the upper and lower boundaries, as well as the left and right boundaries of the lattice, are connected, forming a toroidal topology. The periodic mapping of node coordinates is defined as follows:


$$\begin{array}{c} \;x^{\prime }=((x-1)\mathrm{mod}L)+1,y^{\prime }=((y-1)\mathrm{mod}L)+1. \end{array}$$

This setup ensures that all nodes in the network have an identical number of neighbors $\left|\Omega_{i}\right|=8,\forall i$, thereby completely eliminating the influence of boundary effects on the evolutionary dynamics. Under periodic boundary conditions, no individual lies at a true “system boundary”; all positions are equivalent. This ensures spatial homogeneity and guarantees that the asymptotic behavior observed in simulations represents the infinite-system limit and is robust to lattice size. At any time step *t*, an individual *i* accumulates benefits by engaging in games with all of i*t*s neighbors, and the total benefit $P_{i}(t)$ is calculated as follows:


$$\begin{array}{c} P_{i}(t)=\sum_{j\in \Omega_{i}}\pi (s_{i}(t),s_{j}(t)), \end{array}$$

where $\pi (s_{i},s_{j})$ denotes the benefit obtained from a single game when individual *i* adopts strategy $s_{i}$ and its neighbor *j* adopts strategy $s_{j}$, the value of this benefit is determined by the corresponding game payoff matrix.

After the benefit calculation, all individuals simultaneously update their strategies according to a specific strategy update rule. This study performs a comparative analysis using the Fermi Update Rule and Random Neighbor Unconditional Imitation. Under the Fermi Update Rule, the evolutionary dynamics of the system are simulated on an L×L regular square lattice with periodic boundary conditions to eliminate edge effects. In this study, the evolutionary dynamics are simulated using a random sequential asynchronous updating mechanism. During each Monte Carlo Step (MCS), all individuals on the lattice are randomly permuted, ensuring that each individual is selected exactly once per MCS in a randomized order. When a focal individual *i* is selected, it randomly chooses one individual *j* from its Moore neighborhood $\Omega_{i}$. The payoffs of individuals *i* and *j*, denoted by $P_{i}$ and $P_{j}$, are calculated based on the current strategy configuration at the moment of updating. The strategy update is then applied immediately, and the updated strategy may affect subsequent updates within the same MCS. This study performs a comparative analysis using the Fermi Update Rule and the Random Neighbor Unconditional Imitation rule. Under the Fermi Update Rule, individual *i* adopts the strategy of its neighbor *j* with the transition probability:


$$\begin{array}{c} \;W\left(s_{j}\to s_{i}\right)=\frac{1}{1+\exp\left(\frac{P_{i}-P_{j}}{\kappa }\right)} \end{array}$$

where κ>0 represents the selection intensity parameter, which indicates the level of noise in the individual’s decision-making process. When κ→0, the strategy selection of an individual is entirely based on the benefit, always favoring the strategy with higher benefit (strong selection limit). When κ→∞, the strategy update degenerates into a random walk. Intermediate values of κ are able to simulate bounded rationality, incomplete information, and strategy exploration behaviors observed in real-world decision-making scenarios.

Under the Random Neighbor Unconditional Imitation rule, the same random sequential asynchronous updating scheme is used. When a focal individual *i* is selected, it randomly and uniformly chooses one neighbor *j* from its neighborhood set $\Omega_{i}$ and directly adopts the strategy of *j*, without considering any payoff difference between the two individuals. In the Moore neighborhood, the probability that any given neighbor is selected and imitated is


$$\begin{array}{c} \;W(s_{j}\to s_{i})=\frac{1}{8},\forall j\in \Omega_{i} \end{array}$$

This rule completely eliminates selection pressure linked to fitness and instead models a strategy diffusion process driven by social learning or conformity-based behavior.

Specifically, at each time step t→t+1, all individuals simultaneously update their strategies according to the benefit distribution $P(t)={\left\{P_{i}(t)\right\}}_{i=1}^{N}$ and the strategy configuration $s(t)={\left\{s_{i}(t)\right\}}_{i=1}^{N}$ at time *t*, following the rules described above. $P_{i}(t)$ denotes the payoff of individual *i* at time *t*, and $s_{i}(t)$ denotes the strategy of individual *i* at *t*ime *t*, where *i* = 1,2,......,*N* indexes all individuals in the population. The macroscopic state of the system is characterized by the frequency of *t*he cooperative strategy, denoted by $f_{C}(t)$. Each individual’s strategy belongs to {IC,MC,D}, and $f_{C}(t)$ counts both *IC* and *MC* as cooperators, which is calcula*t*ed as follows:


$$\begin{array}{c} f_{C}(t)=\frac{1}{N}\sum_{i=1}^{n}\delta_{s_{i}(t),C}, \end{array}$$

where $\delta_{s_{i}(t),C}$ denotes the Kronecker delta function, which takes the value 1 when $s_{i}(t)=C$ and 0 otherwise.

## Results

### Theoretical analysis of equilibrium points

Through analytical calculations, it can be shown that the system admits seven equilibrium points, which are given by:


$$\begin{array}{c} \;\begin{array}{c} E_{1}=(0,0,1),E_{2}=(1,0,0),E_{3}=(0,1,0),E_{4}=(\frac{n-k}{b-1},0,\frac{k+b-n-1}{b-1}),\\ E_{5}=(0,-\frac{a+m}{b-\beta -1},\frac{a+m+b-\beta -1}{b-\beta -1}),E_{6}=(\frac{k+\beta -a-c}{\beta -2c},\frac{a-c-k}{\beta -2c},0),E_{7}=(\frac{\varepsilon_{1}}{H},\frac{\varepsilon_{2}}{H},\frac{\varepsilon_{3}}{H}), \end{array} \end{array}$$

where


$$\begin{array}{c} \;\begin{array}{c} H=b\beta -2bc+c^{2}-m^{2}-2mn-n^{2}-\beta +2c,\\ \varepsilon_{1}=G+U+m(m+n),\\ \varepsilon_{2}=G+Q-mn-n^{2},\\ \varepsilon_{3}=Q-U+2bc+\beta -c^{2}-b\beta -2c,\\ G=ab+mb+nb+k-bk-a-m-n,\\ Q=ck+km+kn-cn,\\ U=am+an+\beta k+\beta n-cm-ac. \end{array} \end{array}$$

Specifically, *E*_1_, *E*_2_, and *E*_3_ represent the boundary pure-strategy equilibria, corresponding to states of full defection, full conservative cooperation, and full aggressive cooperation, respectively. *E*_4_, *E*_5_, and *E*_6_ denote the boundary mixed-strategy equilibria, where only two strategies coexist in the population. *E*_7_ is the unique interior equilibrium, characterizing the coexistence of all three strategies (*IC*, *MC*, and *D*).

Due to z=1−x−y, the three-strategy model consisting of conservative cooperation, aggressive cooperation, and defection can be transformed into:


$$\begin{array}{c} \;\begin{array}{ccc} \dot{x} & =f(x,y)= & (b-1)x^{3}+[1-n+(b+m)y-(b-n)(1-y)-(1+c-k)y\\ & & -(n-k)(1-y)-(1+c+m)y]x^{2}+[(1+c-k)y+(n-k)(1-y)\\ & & -(b+m)(1-y)y-((1+\beta -a)y-(a+m)(1-y))y]x,\\ \dot{y} & =g(x,y)= & (b-\beta -1)y^{3}+[1+\beta +m+(b-n)x-(b+m)(1-x)-(1+c-n)x\\ & & -(1+c-a)x+(a+m)(1-x)]y^{2}+[(1+c-a)x-(a+m)(1-x)\\ & & -(b-n)(1-x)x-((1-k)x+(n-k)(1-x))x]y. \end{array} \end{array}$$

By calculating the first-order partial derivatives, the Jacobian matrix of the system of differential equations can be obtained:


$$\begin{array}{c} \;J=\left[\begin{array}{cc} \frac{\partial f(x,y)}{\partial x} & \frac{\partial f(x,y)}{\partial y}\\ \frac{\partial g(x,y)}{\partial x} & \frac{\partial g(x,y)}{\partial y} \end{array}\right], \end{array}$$

where


$$\begin{array}{c} \;\begin{array}{ccc} \frac{\partial f(x,y)}{\partial x} & = & (3b-3)x^{2}+[2-2n+2(b+m)y-2(b-n)(1-y)-2(1+c-k)y\\ & & -2(n-k)(1-y)-2(1+c+m)y]x+(1+c-k)y+(n-k)(1-y)\\ & & -(b+m)(1-y)y-[(1+\beta -a)y-(a+m)(1-y)]y,\\ \frac{\partial f(x,y)}{\partial y} & = & (2b-2c-2)x^{2}+[1+c-n-(b+m)(1-y)+(b+m)y-(1+\beta +m)y\\ & & -(1+\beta -a)y+(a+m)(1-y)]x,\\ \frac{\partial g(x,y)}{\partial x} & = & y(2b-2)x+y[1+c+m-(b-n)(1-y)+(b+m)y-(1+c-k)y\\ & & -(n-k)(1-y)-(1+c+m)y],\\ \frac{\partial g(x,y)}{\partial y} & = & \left(3b-3\beta -3)y^{2}+[2+2\beta +2m+2(b-n)x-2(b+m)(1-x\right)\\ & & -2(1+c-n)x-2(1+c-a)x+2(a+m)(1-x)]y+(1+c-a)x\\ & & -(a+m)(1-x)-(b-n)(1-x)x-[(1-k)x+(n-k)(1-x)]x. \end{array} \end{array}$$

The stability of the system is analyzed by evaluating the determinant and trace of the Jacobian matrix, as detailed below.

1. For the equilibrium point *E*_1_ = (0,0,1), |J|=(k−n)(a+m) and trJ=n−m−a−k. When *k* > *n*, the equilibrium point *E*_1_ is stable.
2. For the equilibrium point *E*_2_ = (1,0,0), |J|=(a−c−k)(n+1−k−b) and trJ=b+c+2k−n−a−1.
  (a) When *a* < *c* + *k* and *n* + 1 < *k* + *b*, the equilibrium point *E*_2_ is unstable.
  (b) When *a* > *c* + *k* and *n* + 1 > *k* + *b*, the equilibrium point *E*_2_ is stable.
3. For the equilibrium point *E*_3_ = (0,1,0), |J|=(a+c−k−β)(a+b+m−β−1) and trJ=2a+b+c+m−2β−k−1.
  (a) When a+c>k+β and a+b+m>β+1, the equilibrium point *E*_3_ is unstable.
  (b) When a+c<k+β and a+b+m<β+1, the equilibrium point *E*_3_ is stable.
4. For the equilibrium point $E_{4}=(\frac{n-k}{b-1},0,\frac{k+b-n-1}{b-1})$, $\left|J\right|=\frac{\left(n-k)[-n^{2}+(\theta_{2}+k)n-\theta_{2}k+\theta_{1}](k+b-n-1\right)}{{\left(b-1\right)}^{2}}$ and $trJ=\frac{k^{2}+(\theta_{2}+b-3n-1)k+2n^{2}+(1-b-\theta_{2})n-\theta_{1}}{b-1}$, where $\theta_{1}=(b-1)(a+m)$ and $\theta_{2}=b-c-m-1$. When $(k+\theta_{2})n-\theta_{2}k>n^{2}-\theta_{1}$ and $k^{2}+(\theta_{2}+b-3n-1)k+2n^{2}<\theta_{1}-(1-b-\theta_{2})n$,the equilibrium point *E*_4_ is stable.
5. For the equilibrium point $E_{5}=(0,-\frac{a+m}{b-\beta -1},\frac{a+m+b-\beta -1}{b-\beta -1})$, $\left|J\right|=\frac{\left[(a+m)(m+\gamma_{2})-\gamma_{1}](a+m)(a+m+b-\beta -1\right)}{{\left(b-\beta -1\right)}^{2}}$ and $trJ=\frac{(a+m)(a+2m+b+\gamma_{2}-\beta -1)-\gamma_{1}}{b-\beta -1}$, where $\gamma_{1}=(k-n)(b-\beta -1)$ and $\gamma_{2}=b+n-c-1$. When $(a+m)(m+\gamma_{2})<\gamma_{1}$ and $(a+m)(a+2m+b+\gamma_{2}-\beta -1)>\gamma_{1}$, the equilibrium point *E*_5_ is unstable.
6. For the equilibrium point $E_{6}=(\frac{k+\beta -a-c}{\beta -2c},\frac{a-c-k}{\beta -2c},0)$, $\left|J\right|=\frac{\left(\mu_{1}c+\mu_{3}-\mu_{4}-c^{2})(a-c-k)(a+c-k-\beta \right)}{{\left(2c-\beta \right)}^{2}}$ and $trJ=\frac{(\mu_{1}+\beta )c+\mu_{5}+\mu_{2}-2c^{2}}{2c-\beta }$, where $\mu_{1}=a+2b+k+m-n-2$, $\mu_{2}=(a-k)(a-k-m-n)$, $\mu_{3}=(n+1-b-k)\beta$, $\mu_{4}=(m+n)(a-k)$, $\mu_{5}=(n+1-a-b)\beta$. When $\mu_{1}c+\mu_{3}>c^{2}+\mu_{4}$ and $(\mu_{1}+\beta )c+\mu_{5}<2c^{2}-\mu_{2}$, the equilibrium point *E*_6_ is unstable.
7. For the equilibrium point $E_{7}=(\frac{\varepsilon_{1}}{H},\frac{\varepsilon_{2}}{H},\frac{\varepsilon_{3}}{H})$, $\left|J\right|=-\frac{\varepsilon_{1}\varepsilon_{2}\varepsilon_{3}}{H^{2}}$ and $trJ=-\frac{(a+c-k-\beta )\varepsilon_{2}+(k-n)\varepsilon_{3}}{H}$. When $H<0,\varepsilon_{1}>0,\varepsilon_{2}<0,\varepsilon_{3}<0,(a+c-k-\beta )\varepsilon_{2}+(k-n)\varepsilon_{3}>0$, the equilibrium point *E*_7_ is stable.

**Fig 1:**
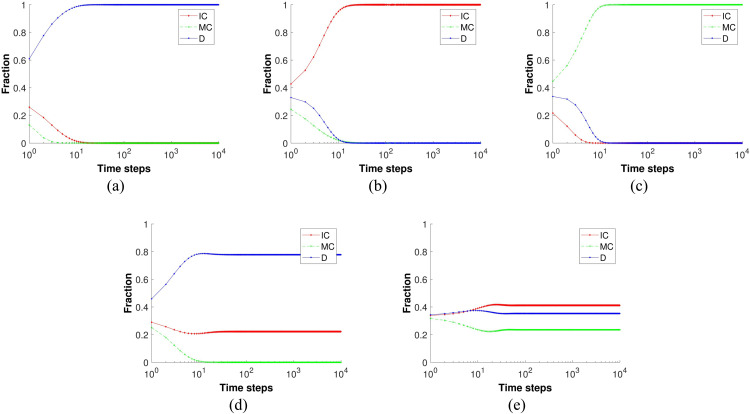
Stable states corresponding to each equilibrium point under different parameter values.

Based on the above analysis, the three-strategy cooperative game-theoretic model proposed in this paper admits five stable equilibrium points. For the equilibrium point *E*_1_, let b=1.8,k=0.5,c=0.3,n=0.2,a=0.8,β=0.1,m=0.7. For the equilibrium point *E*_2_, let b=1.1,k=0.1,c=0.2,n=0.6,a=0.5,β=0.3,m=0.2. For the equilibrium point *E*_3_, let b=1.1,k=0.7,c=0.1,n=0.6,a=0.05,β=0.9,m=0.2. For the equilibrium point *E*_4_, let b=1.9,k=0.2,c=0.3,n=0.4,a=0.1,β=0.1,m=0.3. For the equilibrium point *E*_7_, let b=1.6,k=0.15,c=0.65,n=0.55,a=0.1,β=0.35,m=0.15. Their corresponding equilibrium states are shown in Fig 1.

**Fig 2:**
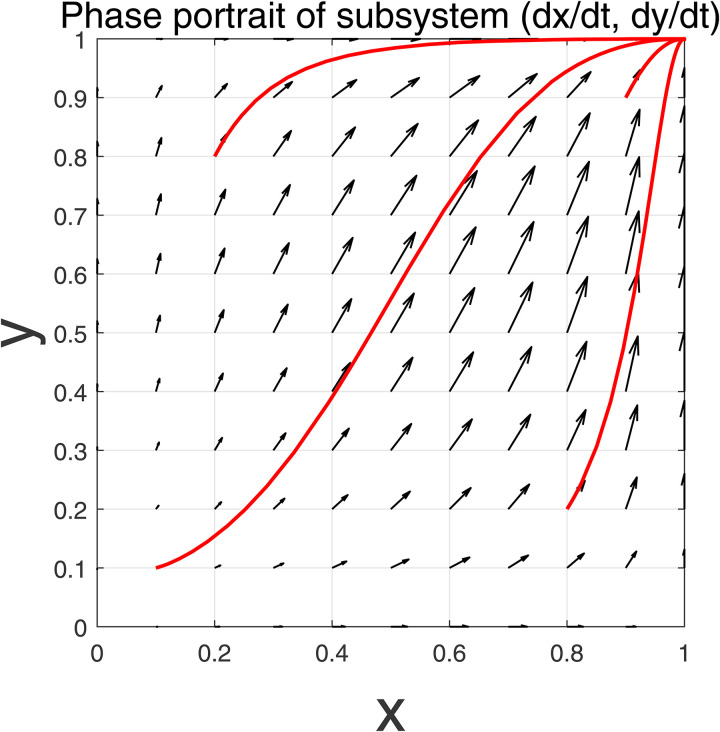
Phase portrait of the two-dimensional subsystem $\left(\dot{x},\dot{y}\right)$ in the x−y plane.

To intuitively demonstrate the dynamic behavior of the system, this paper presents two-dimensional phase portraits of the $\left(\dot{x},\dot{y}\right)$ subsystem under the condition of a fixed variable *z*. Let a=1,b=2,c=0.5,m=0.2,n=0.3,k=0.1,β=0.4,z=0.3. Fig 2 illustrates the vector field distribution and trajectory evolution in the x−y plane. It can be observed that the system converges to the equilibrium point starting from different initial states, which verifies the stability of the system.

**Fig 3:**
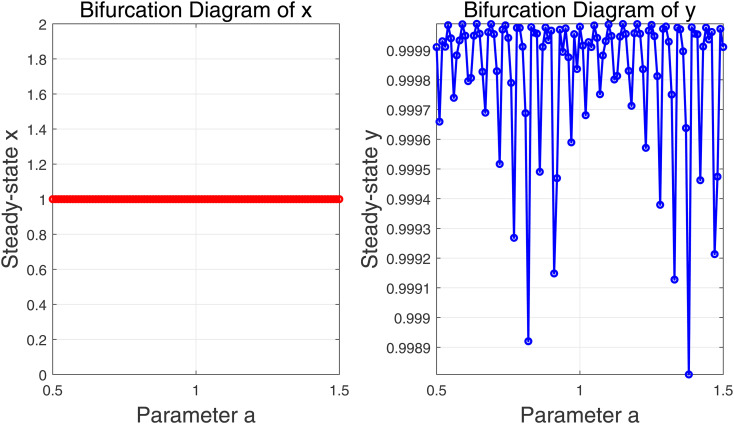
Bifurcation diagrams of the two-dimensional subsystem $\left(\dot{x},\dot{y}\right)$ with respect to parameter *a* with steady-state value of *x* and steady-state value of *y.*

Since the $\left(\dot{x},\dot{y}\right)$ subsystem is two-dimensional and structurally concise, this paper conducts a rigorous bifurcation analysis to reveal how variations in key parameters influence the dynamic behavior of the system. Let b=2,c=0.5,m=0.2,n=0.3,k=0.1,β=0.4,z=0.3. Fig 3 presents the bifurcation diagrams of the two-dimensional subsystem with respect to the key parameter *a*. As shown in Fig 3, the steady-state value of *x* remains constantly at 1 across the entire range of parameter *a*, indicating that the system converges to the boundary equilibrium *x* = 1 regardless of changes in *a*. The steady-state value of *y* fluctuates within a small neighborhood around 1, accompanied by occasional instantaneous drops, reflecting the dynamic adjustment process of the system near the equilibrium point. The bifurcation analysis verifies the stability characteristics of the subsystem and reveals the regulatory effect of parameter *a* on the system’s dynamic behavior.

### Simulation results of infinite well-mixed population model

**Fig 4:**
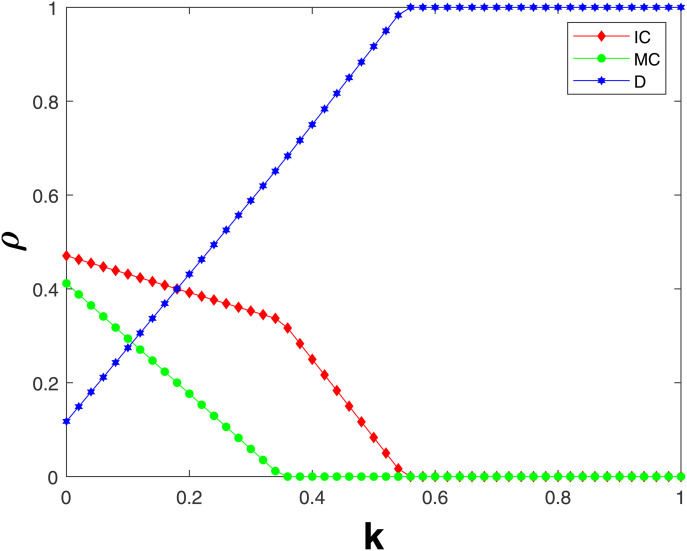
The promoting effect of the identification cost *k* on defectors $\rho_{D}$ and its inhibitory effect on cooperators $\rho_{IC}$, $\rho_{MC}$.

The initial proportions of conservative cooperators, aggressive cooperators, and defectors are all set to 1/3. First, after the system stabilizes, the effects of variations in the identification cost *k* on the proportion of conservative cooperators $\rho_{IC}$, the proportion of aggressive cooperators $\rho_{MC}$ and the proportion of defectors $\rho_{D}$ are presented. Let b=1.6,c=0.65,n=0.55,a=0.1,β=0.35,m=0.15. As shown in Fig 4, when k∈0,0.35, the proportion of conservative cooperators decreases slightly with increasing identification cost, whereas the proportion of aggressive cooperators declines sharply as the identification cost increases. Throughout this range, the proportion of conservative cooperators remains higher than that of aggressive cooperators. At *k* = 0.35, the proportion of aggressive cooperators drops to 0. When *k* > 0.35, the proportion of conservative cooperators decreases markedly and reaches 0 at *k* = 0.55. For k∈0,0.6, the proportion of defectors increases substantially with increasing identification cost, and at *k* = 0.6, the system becomes fully dominated by defectors. From these observations, it can be seen that although an increase in the identification cost reduces the extent to which conservative cooperators are exploited by defectors, defectors are still able to obtain substantial benefits. As a result, the number of cooperators competing with defectors gradually declines. Moreover, a progressive increase in the identification cost is clearly disadvantageous for conservative cooperators, leading to their continuous reduction. This indicates that excessively high identification costs ultimately result in the loss of cooperation.

**Fig 5:**
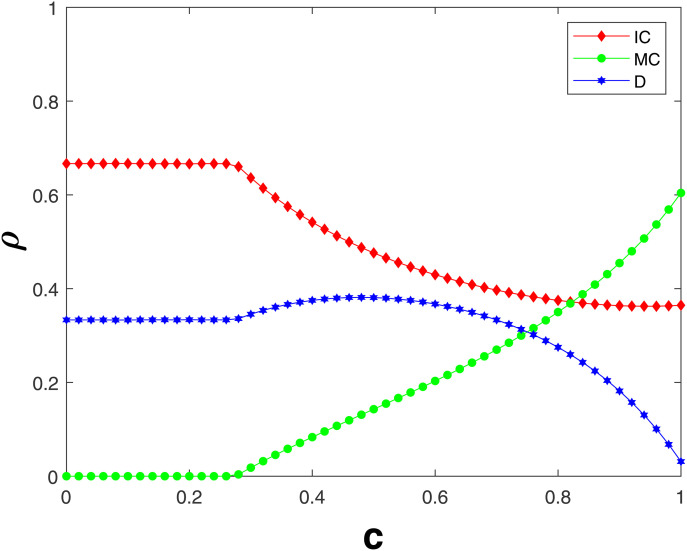
The promoting effect of the cooperative benefit *c* on aggressive cooperators $\rho_{MC}$ and its inhibitory effect on defectors $\rho_{D}$.

Next, let examine the effect of variations in the cooperative benefit *c* on the proportions of conservative cooperators $\rho_{IC}$, aggressive cooperators $\rho_{MC}$, and defectors $\rho_{D}$ after the system reaches a stable state. Let b=1.6,k=0.15,n=0.55,a=0.1,β=0.35,m=0.15. As shown in Fig 5, when c∈0,0.3, the number of aggressive cooperators remains 0, and the proportion of conservative cooperators is higher than that of defectors, with both proportions remaining unchanged. This suggests that, when the increase in benefit is relatively low, conservative cooperators are able to maintain their advantage. When *c* > 0.3, the proportion of aggressive cooperators gradually increases, as the benefit they obtain from cooperating with conservative cooperators continues to rise. Consequently, the aggressive cooperative strategy exhibits a clear advantage and attracts an increasing number of individuals. The proportion of defectors shows a slight increase when the benefit is relatively low, but then decreases progressively as the benefit increases. This indicates that higher cooperative benefits gradually reduce the attractiveness of the defection strategy, leading to a continuous decline in its prevalence. In addition, the proportion of conservative cooperators decreases slowly. This is because, as the benefit continues to increase, the attractiveness of the aggressive cooperative strategy rises rapidly, prompting more individuals to adopt aggressive cooperation, thereby reshaping the overall strategic composition of the population.

**Fig 6:**
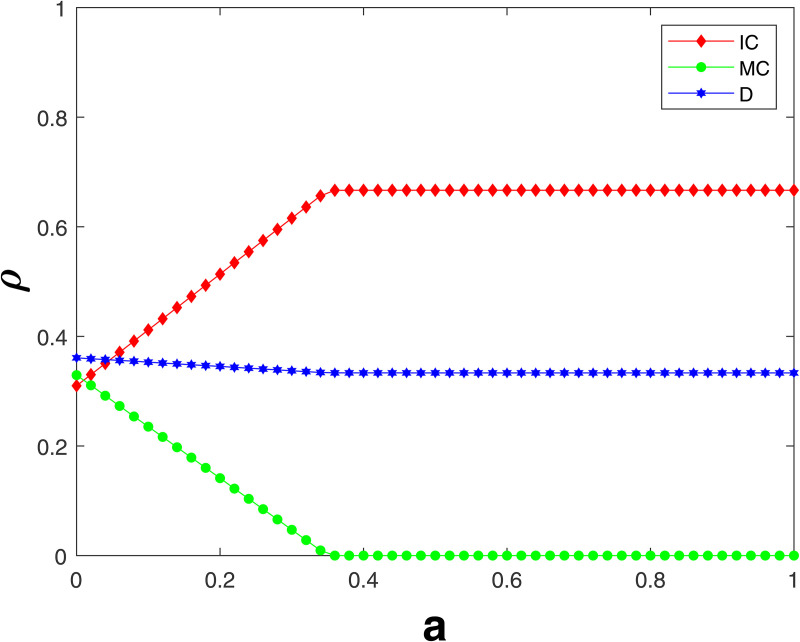
The significant advantage of the aggressive cooperation cost *a* for conservative cooperators $\rho_{IC}$.

Subsequently, let us investigate the effect of variations in the aggressive cooperation cost *a* on the proportions of conservative cooperators $\rho_{IC}$, aggressive cooperators $\rho_{MC}$, and defectors $\rho_{D}$ after the system reaches a stable state. Let b=1.6,k=0.15,c=0.65,n=0.55,β=0.35,m=0.15. As shown in Fig 6, when a∈0,0.35, the proportion of aggressive cooperators decreases gradually with increasing aggressive cooperation cost, and at *a* = 0.35, the proportion of aggressive cooperators drops to 0. When a∈0,0.4, the proportion of conservative cooperators gradually increases, as the higher cost of the aggressive cooperative strategy reduces its attractiveness. In contrast, the lower risk associated with the conservative cooperative strategy leads more individuals to favor adopting it. When *a* > 0.4, the advantage of the conservative cooperative strategy becomes more pronounced, and the proportion of conservative cooperators approaches a stable value. In Fig 6, the proportion of defectors remains largely unchanged and stays at a relatively low level. This is because the variation in the aggressive cooperation cost primarily impacts the distribution between conservative and aggressive cooperators, with minimal effect on defectors. Therefore, the defection strategy does not dominate the population but maintains a stable proportion. This indicates that changes in the aggressive cooperation cost mainly alter the overall strategic composition by affecting the proportions of the two cooperative strategies.

**Fig 7:**
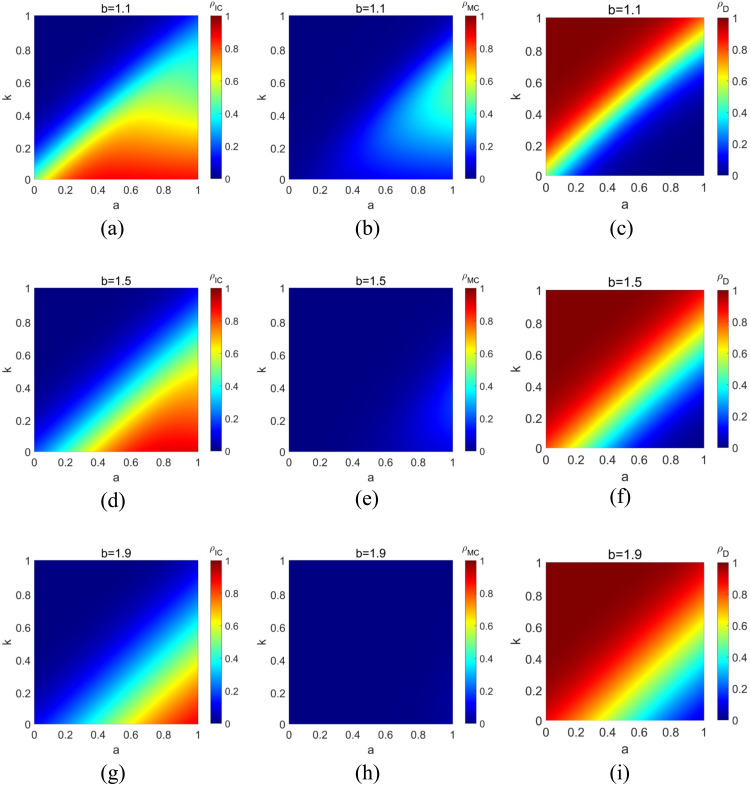
Heat maps on the diminishing inhibitory effect of high aggressive cooperation cost *a* and low identification cost *k* on the proportion of defectors with increasing temptation to defect *b.* Panels (a), (d), and (g) represent the impact of parameter variations on $\rho_{IC}$. Panels (b), (e), and (h) represent the impact of parameter variations on $\rho_{MC}$. Panels (c), (f), and (i) represent the impact of parameter variations on $\rho_{D}$.

The above analysis validates the effect of independent parameter variations on strategy selection. However, the interactions and inherent relationships between parameters are equally important. Therefore, in the following analysis, a heatmap of the bivariate interaction effects on strategy selection is presented. First, let us analyze the heatmap between the aggressive cooperation cost *a* and the identification cost *k*, with c=0.65,n=0.55,β=0.35,m=0.15. As shown in Fig 7(a)–7(c), the proportion of conservative cooperators increases with the aggressive cooperation cost *a* and decreases with increasing identification cost. This indicates that a higher aggressive cooperation cost enhances the relative benefit of conservative cooperators within the set of cooperative strategies, thereby encouraging more individuals to adopt the conservative cooperative strategy when the cost of aggressive cooperation is high. Although an increase in the identification cost strengthens the ability of conservative cooperators to resist exploitation by defectors, defectors are still able to obtain substantial benefits. This, in turn, partially reduces the benefit of conservative cooperators and consequently weakens the attractiveness of the conservative cooperative strategy. In addition, as the temptation to defect increases, the proportion of conservative cooperators is suppressed under conditions of low aggressive cooperation cost and high identification cost. As shown in Fig 7(a)–7(c), the proportion of aggressive cooperators increases gradually with the aggressive cooperation cost only when *b* = 1.1. Additionally, with increasing identification cost, the proportion first increases, then decreases. When *b* = 1.5 and *b* = 1.9, there is no significant change in the proportion of aggressive cooperators. This suggests that variations in the aggressive cooperation cost and identification cost do not have a significant impact on the proportion of aggressive cooperators. As shown in Fig 7(g)–7(i), the proportion of defectors decreases with increasing aggressive cooperation cost *a* and increases with rising identification cost. This is because a higher aggressive cooperation cost may drive the cooperative population to favor the conservative cooperative strategy, thereby reducing the benefit obtained by defectors and consequently leading to a decline in their proportion. An increase in the identification cost reduces the benefit of a subset of conservative cooperators, while the incremental rise in identification cost does not substantially affect the benefit of defectors. As a result, some conservative cooperators are inclined to switch to the defection strategy. Overall, as the temptation to defect increases, the proportion of defectors is less strongly suppressed under conditions of high aggressive cooperation cost and low identification cost.

**Fig 8:**
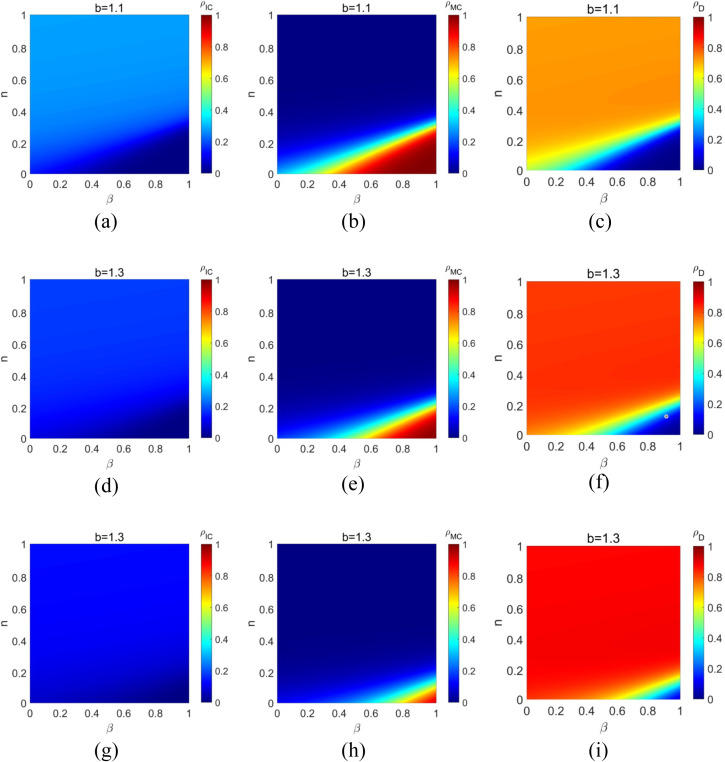
Heat maps the inhibitory effect of higher aggressive benefit β and lower conservative benefit *n* on the proportion of defectors. Panels (a), (d), and (g) represent the impact of parameter variations on $\rho_{IC}$. Panels (b), (e), and (h) represent the impact of parameter variations on $\rho_{MC}$. Panels (c), (f), and (i) represent the impact of parameter variations on $\rho_{D}$.

Subsequently, let analyze the heatmap of the interaction between the aggressive benefit β and the conservative benefit *n*, with *k* = 0.15, *c* = 0.05, *a* = 0.1, *m* = 0.05. As shown in Fig 8(a)–8(c), only when *b* = 1.1 does the proportion of conservative cooperators exhibit a decreasing trend with decreasing conservative benefit and increasing aggressive benefit; however, the magnitude of this change is not significant. This indicates that variations in the conservative benefit *n* and the aggressive benefit β do not have a significant impact on the proportion of conservative cooperators. As shown in Fig 8(d)–8(f), the proportion of aggressive cooperators is positively correlated with the aggressive benefit and negatively correlated with the conservative benefit. This is because an increase in the conservative benefit enhances the attractiveness of the conservative cooperative strategy, thereby inducing some aggressive cooperators to switch to conservative cooperation. Meanwhile, as the temptation to defect increases, the proportion of aggressive cooperators is suppressed under conditions of high aggressive benefit and low conservative benefit. As shown in Fig 8(g)–8(i), the proportion of defectors decreases with increasing aggressive benefit and increases with increasing conservative benefit. This is because a higher aggressive benefit enhances the attractiveness of cooperative strategies, thereby motivating some defectors to switch to cooperation. In contrast, an increase in the conservative benefit implies that, from the perspective of defectors, the penalty imposed by conservative cooperators during interactions is relatively small compared to the gains from defection. When the conservative benefit is high, individuals perceive the losses incurred from interacting with defectors as negligible; consequently, some individuals believe that the defection strategy allows them to obtain defection benefits at a low cost, leading to a higher willingness to adopt defection. Overall, the proportion of defectors is suppressed under conditions of high aggressive benefit and low conservative benefit.

### Simulation results of spatiotemporal evolution in structured populations

In structured populations, all simulations are performed on a 100*100 square lattice. In addition, simulations on larger lattices are also conducted to rule out finite-size effects, and similar results are obtained. The initial population proportions in the structured population are set to be identical to those in the infinite well-mixed population. Periodic boundary conditions are used to avoid boundary bias and ensure equal neighborhood size and interaction opportunities for all individuals, making the system spatially homogeneous. If fixed boundaries were used instead, individuals at edges would have fewer interactions and lower payoffs, leading to distorted strategy survival and evolution. Periodic boundaries therefore ensure that our results reflect genuine strategy dynamics rather than artificial boundary artifacts.

**Fig 9:**
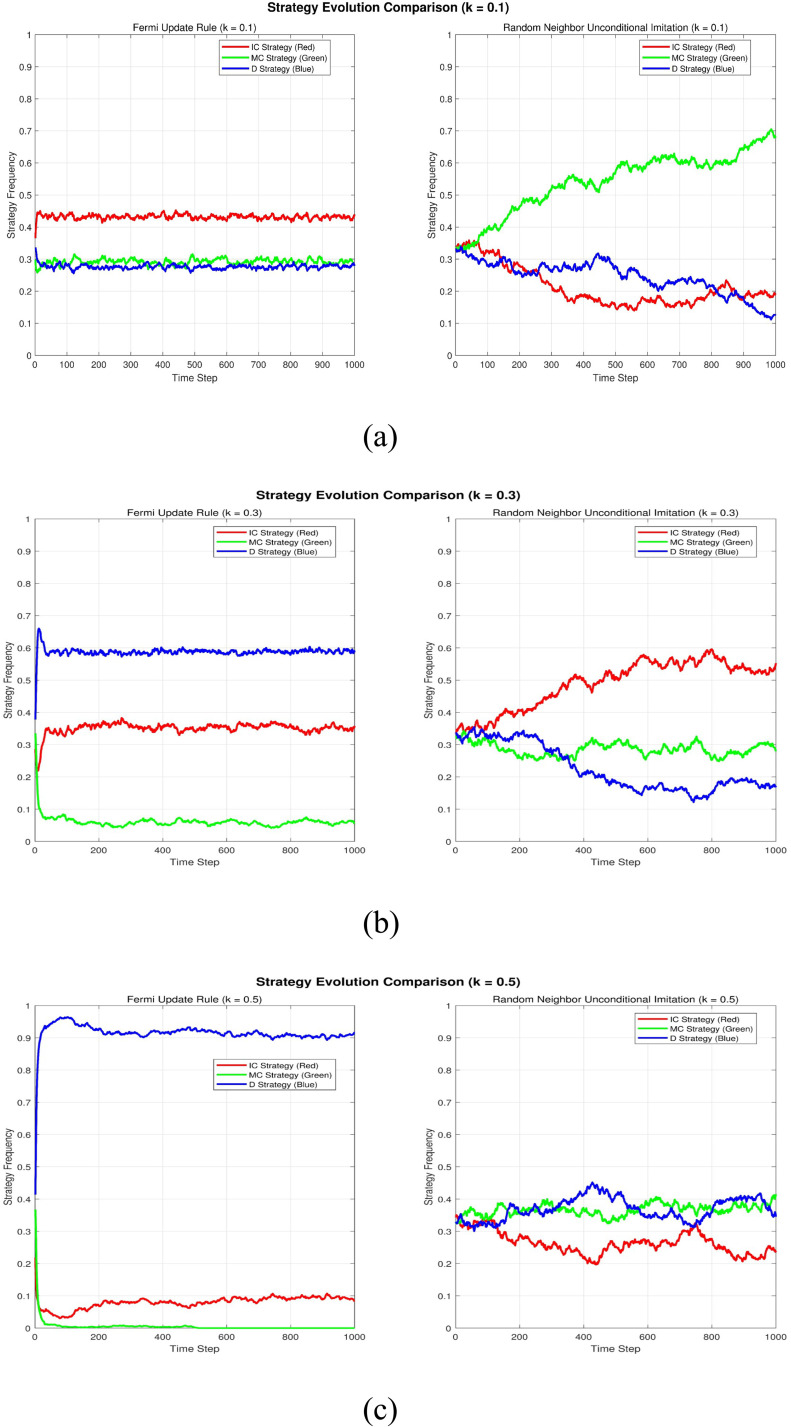
Population distribution proportions of different strategies at k∈0.1,0.3,0.5 and the phased evolutionary characteristics under the random neighbor unconditional imitation update rule.

First, let b=1.6,a=0.1,c=0.65,n=0.55,β=0.35,m=0.15. The population distributions of the three strategies for k∈0.1,0.3,0.5 are shown in Fig 9. Under the Fermi updating rule, when *k* = 0.1, the proportion of conservative cooperators remains consistently high compared with that of aggressive cooperators and defectors. When *k* = 0.3, the proportion of defectors becomes relatively high, accompanied by a decline in the proportion of cooperators. When *k* = 0.5, the proportion of defectors rises to between 0.9 and 1, the proportion of conservative cooperators drops below 0.1, and aggressive cooperators disappear. These results indicate that when the identification cost increases beyond a certain threshold, the benefit advantage of conservative cooperators is no longer maintained, leading to a reduction in their population share. Although an increase in the identification cost can partially protect conservative cooperators from exploitation by defectors, defectors are still able to obtain relatively high benefits, resulting in a gradual increase in their proportion. Under the Random Neighbor Unconditional Imitation updating rule, when *k* = 0.1,the proportion of aggressive cooperators remains at a relatively high level. When *k* = 0.3, the proportion of conservative cooperators becomes the highest. When *k* = 0.5, the three populations exhibit a mixed and interwoven distribution. This is because, under the Random Neighbor Unconditional Imitation updating rule, individuals do not evaluate the benefits of the three strategies but instead blindly imitate the decisions of neighboring individuals when making their own strategy choices. Consequently, the initial spatial distribution of strategies has a significant influence on the population proportions of the different strategies.

**Fig 10:**
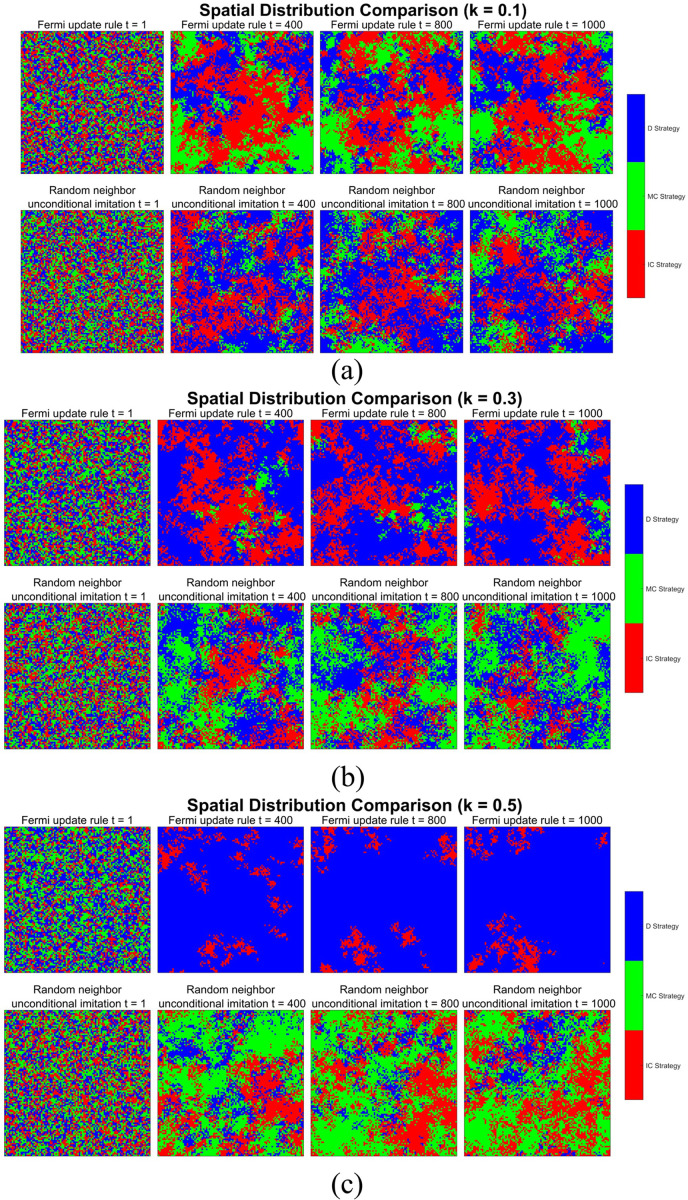
The spatial snapshots of population distributions for different strategies at k∈0.1,0.3,0.5.

Next, let us examine the spatial distribution snapshots, as shown in Fig 10. Under the Fermi updating rule, the snapshots reveal that when *k* = 0.1, the population of conservative cooperators gradually expands outward, while aggressive cooperators and defectors also maintain certain proportions. When *k* = 0.3, defectors gain a clear advantage in spatial distribution, and the populations of both cooperative strategies continuously shrink. When *k* = 0.5, only defectors and conservative cooperators remain in the spatial distribution, with aggressive cooperators disappearing completely. These results indicate that although an increase in the identification cost can, to some extent, help conservative cooperators reduce exploitation by defectors, it exerts a strong negative impact on cooperative strategies overall, ultimately allowing defectors to dominate the population. From the spatial distribution snapshots under the Random Neighbor Unconditional Imitation updating rule, it can be observed that when *k* = 0.1, aggressive cooperators initially occupy a dominant position, and over time this leads neighboring individuals to unconditionally adopt the aggressive cooperative strategy. When *k* = 0.3, conservative cooperators begin to gain dominance and spread over a wide area, while the other two populations, due to their relatively dispersed distributions, maintain certain proportions. When *k* = 0.5, the three strategies are initially distributed relatively evenly, and as time evolves, all three strategies continue to coexist at stable proportions.

**Fig 11:**
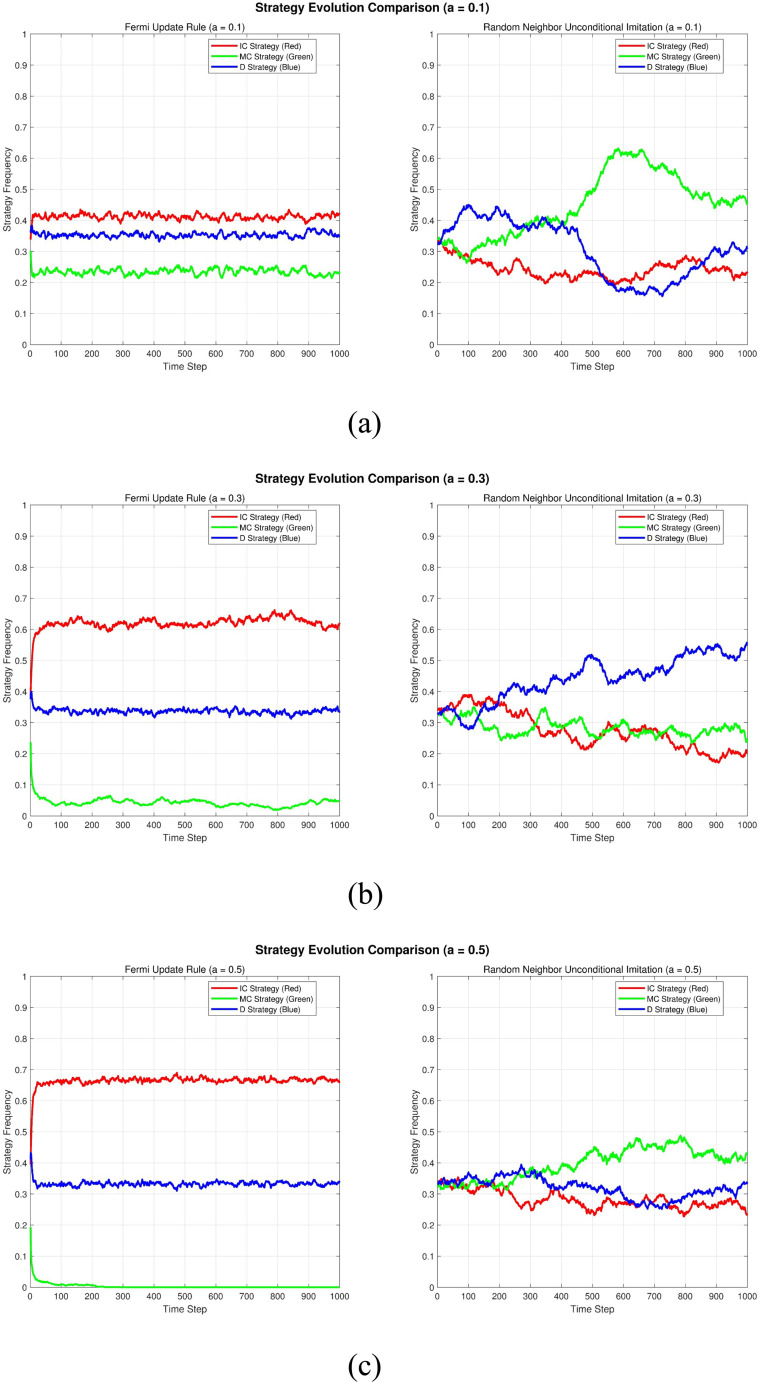
Population distribution proportions of different strategies at a∈0.1,0.3,0.5 and the phased evolutionary characteristics under the random neighbor unconditional imitation update rule.

Subsequently, let b=1.6,k=0.15,c=0.65,n=0.55,β=0.35,m=0.15;. The population distributions of the three strategies for a∈0.1,0.3,0.5 are shown in Fig 11. Under the Fermi updating rule, when *a* = 0.1, the proportion of conservative cooperators is relatively high, and the proportions of the three strategies do not differ substantially. When *a* = 0.3, the proportion of conservative cooperators increases to above 0.6, while the proportion of aggressive cooperators decreases to below 0.1. When *a* = 0.5, aggressive cooperators disappear from the spatial system, whereas the proportion of conservative cooperators remains at a relatively high level. These results indicate that, as the aggressive cooperation cost increases, the attractiveness of the aggressive cooperative strategy gradually declines, and individuals tend to prefer the lower-risk conservative cooperative strategy. Although the defection strategy does not become dominant in the population, it maintains a stable proportion. Overall, changes in the aggressive cooperation cost primarily alter the overall strategic composition by affecting the population shares of the two cooperative strategies. Under the Random Neighbor Unconditional Imitation updating rule, participants do not engage in strategic evaluation but instead simply imitate the choices of nearby individuals. Consequently, individuals choosing the three different strategies are randomly and non-uniformly distributed in space. The final proportions of individuals choosing these strategies exhibit fluctuations in response to changes in parameter *a*. Essentially, the variation in *a* drives the strategy selection of every individual within the population.

**Fig 12:**
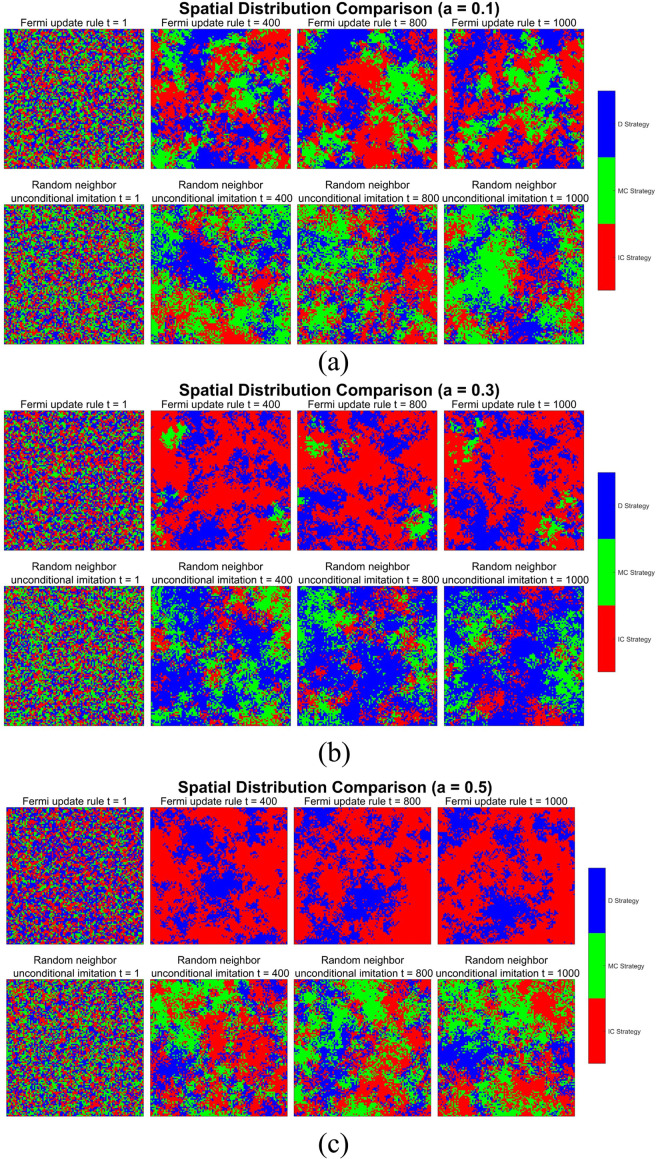
The spatial snapshots of population distributions for different strategies at a∈0.1,0.3,0.5.

Next, let analyze the spatial distribution snapshots, as shown in Fig 12. Under the Fermi updating rule, the snapshots indicate that when *a* = 0.1, conservative cooperators continuously expand over time, while aggressive cooperators and defectors maintain certain proportions. When *a* = 0.3, conservative cooperators occupy a large area, defectors hold a small fraction of the territory, and aggressive cooperators are present at a very low proportion. When *a* = 0.5, the spatial system consists mainly of conservative cooperators with only a small proportion of defectors, and aggressive cooperators disappear completely. These results indicate that an increase in the aggressive cooperation cost encourages more individuals to adopt the more cautious conservative cooperative strategy, thereby promoting the prevalence of conservative cooperation within the population, while having little impact on defectors. From the spatial distribution snapshots under the Random Neighbor Unconditional Imitation updating rule, it can be observed that, due to a “herd” effect, aggressive cooperators gradually gain dominance in the population when *a* = 0.1 because of their large initial spatial coverage. When *a* = 0.3, defectors gain an advantage owing to their more dispersed spatial distribution. When *a* = 0.5, the three strategies, starting from relatively uniform initial distributions, gradually evolve toward a stable coexistence pattern.

**Fig 13:**
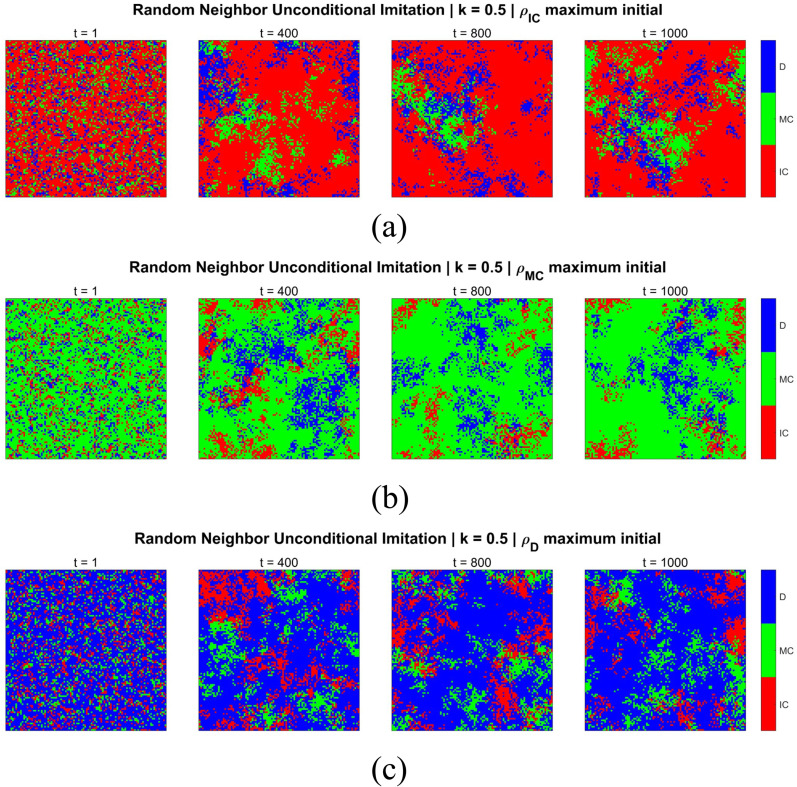
Spatial distributions of strategy selection over time under different initial proportions at *k* = 0.5. Panels (a), (b), and (c) correspond to initial proportions of $\rho_{IC}$=0.7, $\rho_{MC}$=0.7, and $\rho_{D}$=0.7, respectively.

Finally, to examine the relationship between individual strategy selection and initial conditions under the Random Neighbor Unconditional Imitation rule, we considered the case of an identification cost of *k* = 0.5 and separately set the initial proportion of conservative cooperators $\rho_{IC}$, aggressive cooperators $\rho_{MC}$, and defectors $\rho_{D}$ to 0.7. As shown in Fig 13, the larger the initial proportion of a given strategy, the more individuals ultimately adopt that strategy. In other words, the initial strategy distribution continues to influence and shape the final population configuration through local-neighborhood diffusion, highlighting the decisive role of initial conditions under the Random Neighbor Unconditional Imitation rule.

## Conclusion

This study introduces conservative cooperation and aggressive cooperation as two cost-dependent extensions of conventional cooperative behavior, and investigates their roles in the evolution of cooperation in well-mixed and structured populations. Conservative cooperators reduce potential losses from defectors by paying an identification cost, whereas aggressive cooperators obtain enhanced cooperative benefits by paying an additional cooperation cost. By incorporating these two mechanisms, this work provides a more differentiated framework for understanding how cooperation can emerge and persist under heterogeneous cost-benefit conditions.

The results show that cooperation is shaped by the combined effects of cost structure, cooperative benefit, and population organization. In well-mixed populations, lower identification costs and higher cooperative benefits promote cooperation within specific parameter ranges. In structured populations under random neighbor unconditional imitation update rule, local interactions further reshape the evolutionary process, leading to stage-dependent shifts in population dominance rather than a fixed globally dominant strategy. Conservative cooperation becomes advantageous when the identification cost remains sufficiently low, while aggressive cooperation promotes cooperation only when its additional cost does not exceed the benefit it generates. These findings suggest that stable cooperation requires a balance between reducing exploitation risk and maintaining sufficient cooperative returns.

The implications of this study extend beyond theoretical evolutionary games. In organizational collaboration, public-resource governance, platform regulation, and socio-economic networks, actors often face a trade-off between screening unreliable partners and investing in stronger cooperation. The results indicate that reducing identification or screening costs, together with maintaining reasonable cooperative incentives, may help stabilize cooperation in complex adaptive systems. They also show that spatial structure does not universally promote cooperation; instead, its effect depends on the underlying cost-benefit configuration and local interaction patterns.

Several limitations remain. The model uses simplified payoff matrices and fixed strategy types, which helps isolate the core mechanisms but cannot fully capture real-world decision-making complexity. The structured population is represented by a regular lattice, which differs from heterogeneous social, ecological, and economic networks. In addition, the current framework does not incorporate adaptive learning, reputation, punishment, partner selection, or institutional intervention.

Future research can extend this work by constructing more realistic and heterogeneous payoff matrices, testing the model on small-world, scale-free, multilayer, and temporal networks, and incorporating adaptive costs, learning rules, reputation effects, and partner-selection mechanisms. Another important direction is to develop optimal control strategies to examine how external incentives, regulation, or institutional design can guide the system toward more stable and socially desirable cooperative states.
